# Functional identification of lncRNAs in sweet cherry (*Prunus avium*) pollen tubes via transcriptome analysis using single-molecule long-read sequencing

**DOI:** 10.1038/s41438-019-0218-3

**Published:** 2019-12-01

**Authors:** Yang Li, Chuanbao Wu, Chunsheng Liu, Jie Yu, Xuwei Duan, Wenqi Fan, Jing Wang, Xiaoming Zhang, Guohua Yan, Tianzhong Li, Kaichun Zhang

**Affiliations:** 1 Laboratory of Fruit Cell and Molecular Breeding, China Agricultural University, Beijing, China; 2 Institute of Forestry and Pomology, Beijing Academy of Agriculture and Forestry Sciences, Beijing, China; 3 Key Laboratory of Biology and Genetic Improvement of Horticultural Crops (North China), Ministry of Agriculture, Beijing, China; 4 Beijing Engineering Research Center for Deciduous Fruit Trees, Beijing, China

**Keywords:** Non-coding RNAs, Reproductive biology

## Abstract

Sweet cherry (*Prunus avium*) is a popular fruit with high nutritional value and excellent flavor. Although pollen plays an important role in the double fertilization and subsequent fruit production of this species, little is known about its pollen tube transcriptome. In this study, we identified 16,409 transcripts using single-molecule sequencing. After filtering 292 transposable elements, we conducted further analyses including mRNA classification, gene function prediction, alternative splicing (AS) analysis, and long noncoding RNA (lncRNA) identification to gain insight into the pollen transcriptome. The filtered transcripts could be matched with 3,438 coding region sequences from the sweet cherry genome. GO and KEGG analyses revealed complex biological processes during pollen tube elongation. A total of 2043 AS events were predicted, 7 of which were identified in different organs, such as the leaf, pistil and pollen tube. Using BLASTnt and the Coding-Potential Assessment Tool (CPAT), we distinguished a total of 284 lncRNAs, among which 154 qualified as natural antisense transcripts (NATs). As the NATs could be the reverse complements of coding mRNA sequences, they might bind to coding sequences. Antisense transfection assays showed that the NATs could regulate the expression levels of their complementary sequences and even affect the growth conditions of pollen tubes. In summary, this research characterizes the transcripts of *P. avium* pollen and lays the foundation for elucidating the physiological and biochemical mechanisms underlying sexual reproduction in the male gametes of this species.

## Introduction

During plant sexual reproduction, pollen plays a vital role in the transportation of male gametes to the ovule, and the cellular and regulatory mechanisms underlying pollen tube elongation have been an ongoing focus of investigation^1^. Pollen tube growth is an attractive model system involving a number of physiological and biological processes, such as signal transduction and polarized cell growth^2,3^. However, the mechanism of complicated process of pollen tube elongation remains largely unknown. RNA sequencing of pollen tubes is a useful method for obtaining global transcriptional information as a starting point for pollen-related research. Short-read sequence data from pollen tubes have been obtained for a variety of species^4–7^ in studies that have focused mainly on the differential expression of genes in different varieties of pollen. To further investigate the molecular mechanism of pollen tube growth, relative entire transcriptome data and additional transcript isoforms are necessary; single-molecule long-read sequencing (PacBio sequencing) technology is well suited for addressing such questions^8^.

Sweet cherry is one of the most popular fruits in the world and is highly nutritious, containing beneficial levels of dietary fiber, ascorbic acid, and carotenoids^9^. As the development of the mature fruit depends on double fertilization, viable pollen is important for sweet cherry production. The sweet cherry genome, with an assembled sequence of 272.4 Mb, was released in 2017^10^; this genome was based on short reads from pollen-containing organs, such as flowers and anthers, sequenced using the Illumina platform^10,11^. However, sequencing data specifically from sweet cherry pollen tubes are lacking, especially global data based on long-read sequencing technology. Recently, an increasing number of studies have focused on the differences in transcribed isoforms (such as AS and noncoding RNA), which have emerged as important regulators of gene expression and plant development^12^. Long-read sequencing technology is a way to obtain relative whole-transcriptome data and additional transcripts/different transcribed isoforms (such as those resulting from AS or noncoding RNA) and is useful for investigating pollen tube growth.

AS is a common biological phenomenon whereby one coding gene can produce multiple proteins^13^. In plants, AS has been found to occur in 61.2%, 42.4%, and 56.4% of genes in *Arabidopsis thaliana*, rice (*Oryza sativa*), and maize (*Zea mays*), respectively^14–16^, and plays important roles in crucial physiological processes. For example, the flower repressor gene *FLOWERING LOCUS M* in *Arabidopsis thaliana* has two isoforms, *FLM-β* and *FLM-δ*, whose production is increased by low and high ambient temperature, respectively, and these isoforms regulate flowering time^17^. The maize gene *ZmbohB* has two isoforms that are differentially expressed in response to various abiotic conditions such as cold, heat, and salt stress^18^. AS has been shown to be involved in the regulation of vernalization, the circadian clock, and biotic and abiotic stress responses in several species^17,18^. However, relatively little research on AS has been performed in the Rosaceae or in sweet cherry in particular.

Setting aside the direct effects of AS in plants, long noncoding RNAs (lncRNAs), tentatively defined as RNAs longer than 200 bp with low coding potential, are also key molecular regulators of plant biology and physiology^19^. The history of lncRNA research dates back more than 25 years, to investigations of *XIST* in humans^20^. Due to their low coding potential and the fact that they sometimes share small modifications with coding mRNAs, lncRNAs were originally regarded as transcriptional noise from the genome and were only later determined to be functional themselves. In plants, lncRNAs can be classified into two groups, polyadenylated and nonpolyadenylated^21^; the nonpolyadenylated lncRNAs are a recently identified group that was first found in rice and *A. thaliana*^22,23^, and the polyadenylated lncRNAs, some of which are transcribed by RNA polymerase II, consist of three groups: long intergenic noncoding RNAs (ncRNAs), intronic noncoding RNAs (incRNAs), and natural antisense transcripts (NATs)^21^. NATs are transcribed in the opposite direction of protein-coding genes and are widespread in animals and plants^21^. LncRNAs are involved in several aspects of plant biology, including transcriptional regulation, histone modification, posttranscriptional regulation, and small RNA production^24–27^. These processes can affect plant vernalization, light signal transduction, root development, and abiotic and biotic stress resistance in different ways^26,28–31^. *LDMAR* is a crucial lncRNA from rice that contributes to pollen grain development when its transcript level exceeds a threshold value^32^. Above all, lncRNAs have been identified and implicated in plant growth and stress responses. However, research on lncRNAs in the pollen of sweet cherry is still lacking.

In this study, to explore the function of transcripts in gene regulation in the pollen of sweet cherry, we employed single-molecule long-read sequencing to obtain a broad set of information about the sweet cherry pollen tube transcriptome. In this manuscript, transposons, isoform types, AS, and lncRNA were analyzed. The information obtained in this study will facilitate further studies on pollen tube growth in sweet cherry.

## Results

### Preliminary analysis of the full-length transcriptome by single-molecule sequencing

**Fig. 1:**
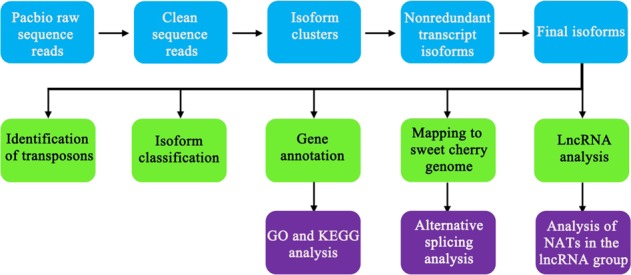
Basic pipeline for PacBio sequencing of sweet cherry pollen tubes. The analysis consists of three parts, starting with sequence filtering and clustering of the raw data to generate the final isoforms (blue boxes). To obtain global information, five types of bioinformatics analysis were performed (green boxes). GO and KEGG analysis, AS, and NAT analysis were based on the analysis of some of the ‘green boxes’ (analyses in purple boxes).

To obtain as many different pollen tube-derived transcripts as possible, we collected and cultured pollen grains from three different sweet cherry cultivars, ‘Rainier’, ‘Lapins’, and ‘21-21’. The germination rates were 81.2%, 79.8%, and 68.5%, respectively. We then extracted RNA for library construction. Multiple size-fractionated libraries were produced using a SageELF device (Supplementary Fig. 1). Each barcoded SMRTBell library was processed on the PacBio RS II platform with two SMRT cells. Once the raw data were obtained, adapters and artificial sequences were removed (Fig. 1). After quality control (Supplementary Fig. 2d–f), reads were clustered and prepared for analysis (Fig. 1). We obtained a total of 465,607 high-quality reads of inserts from six SMRT cells (Table 1). The mean read lengths for the libraries with each size range were 1920 bp for the 1–2 kb library, 2647 bp for the 2–3 kb library, and 3811 bp for the 3–6 kb library (Supplementary Fig. 2a, b, c). The proportions of full-length nonchimeric reads (FL reads) and poly-A reads were 43.1% and 57.0%, respectively, in the 1–2 kb library; 40.2% and 53.9% in the 2–3 kb library; and 2.3% and 26.5% in the 3–6 kb library (Table 1, Supplementary Table 1). As the FL reads are useful in transcript assembly, we also determined the length distribution statistics of the FL reads in different sizes of libraries (Supplementary Fig. 3). We found that the average lengths in the 1–2 kb, 2–3 kb, and 3–6 kb libraries were 1592 bp, 2427 bp, and 3594 bp, respectively (Supplementary Table 1).

**Table 1:** Basic data from PacBio sequencing.

Sample	Cell	Reads of insert	Mean length of insert	Consensus isoforms	Polished isoforms
1–2 kb	2	143452	1920	14724	9096
2–3 kb	2	163008	2647	15195	6385
3–6 kb	2	159147	3811	2706	928

Based on these results, consensus isoforms were called to obtain nonredundant transcript isoforms by using PacBio SMRT software. In total, 32,625 isoforms were regarded as consensus isoforms, and the isoforms were then polished using Quiver. A total of 5628, 8810, and 1778 highly polished isoforms were filtered out of the 1–2 kb, 2–3 kb, and 3–6 kb libraries, respectively (Table 1). We ultimately obtained 16,409 highly polished isoforms and subjected them to bioinformatics analysis. Because of the technical limitations of PacBio sequencing, single-base insertions or deletions could exist in some isoforms. Sequence data from the Illumina platform are helpful for correcting PacBio data^33^. Short-read data for sweet cherry obtained using the Illumina platform were released along with the V1.0 genome (http://cherry.kazusa.or.jp/). We therefore corrected our data based on these short-read data before performing further analyses, including transposon and lncRNA prediction, isoform classification, gene annotation, and AS analysis (Fig. 1).

### Isoform characterization and gene annotation

**Fig. 2:**
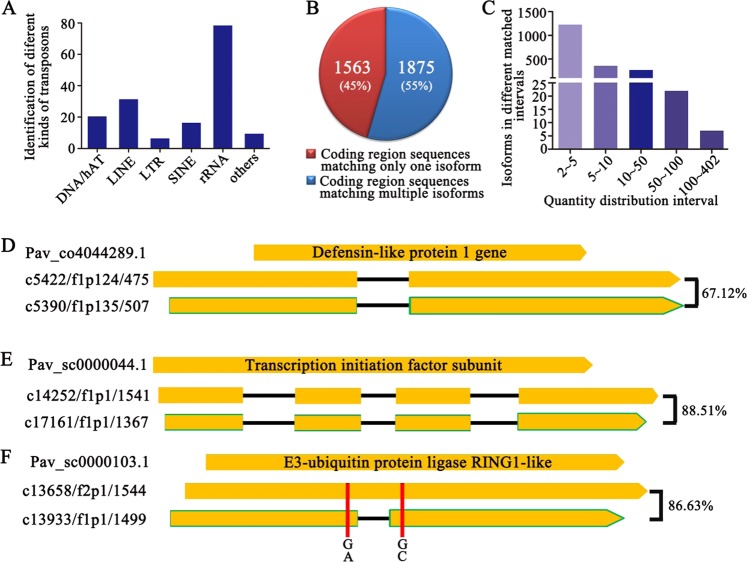
Isoform classification. **a** Identification and classification of transposable elements in sequences identified by PacBio sequencing. **b** Isoform classification by comparison with the coding region sequence file from the sweet cherry genome. **c** Distribution of coding region sequences matching more than one nonredundant transcript. **d** Two isoforms of c5390/f1p135/507 and c5422/f1p124/475 matched the *Pav_co4044289.1* gene, and the black lines represent the unmatched area. **e** Two isoforms of c14252/f1p1/1541 and c17161/f1p1/1367 matched *Pav_sc0000044.1*, and the black lines represent the unmatched area. **f** Two isoforms of c13658/f2p1/1544 and c13933/f1p1/1499 matched *Pav_sc0000103.1*, the black lines represent the unmatched area, and the vertical red lines represent SNPs between two isoforms.

After obtaining the set of 16,409 nonredundant isoforms, we next identified the transposable elements (TEs) among them using RepeatMasker software, which classified 166 isoforms as TEs based on features such as repeat sequences. The TEs were classified into six groups: 21 were classified as a type of DNA/hat, 32 as long interspersed nuclear elements (LINEs), 7 as long terminal repeat elements (LTRs), 17 as short interspersed nuclear elements (SINEs), and 79 as rRNA elements. There were also 10 putative TEs of other types (Fig. 2a).

Having excluded the 166 TEs, we subjected the remaining 16,243 isoforms to further analysis. We attempted to classify the isoforms by comparing them with the genome of sweet cherry (http://cherry.kazusa.or.jp/). First, we extracted coding region sequences (exons + introns) from the sweet cherry genome using a Python script. A total of 43,673 different nucleotide coding sequences were obtained, and isoforms from the PacBio results were compared with these sequences. Among the 43,673 coding region sequences, 3438 were found to match isoforms identified by PacBio sequencing with a cut-off e-value < 10^−10^. Not all the isoforms from long-read sequencing matched the genomic sequences; 295 of the 16,409 isoforms did not match any coding region sequences. Then, NCBI BLAST analysis was used to identify the 295 unmatched isoforms, 166 of which were considered to be cherry virus sequences. The other 129 isoforms were annotated as predicted sequences, including 110 coding sequences and 19 ncRNAs. These 129 isoforms can be found in different locations of sweet cherry chromosomes; however, they were missing in the sweet cherry transcript database (https://www.rosaceae.org/), which implied that they might be newly reported transcripts in sweet cherry pollen.

We divided the matched isoforms into two groups: coding region sequences that matched only one isoform (1563 members, or 45%) and coding region sequences that matched more than one isoform (1875 members, or 55%) (Fig. 2b). In the second group, genetic diversity might be the reason that coding region sequences matched a few PacBio isoforms, with the number usually ranging from 2–5 and falling below 50 in most cases (Fig. 2c). We found that there 7 coding region sequences matched more than 100 isoforms, which may be due to multiple reasons. For example, a large number of homologous genes existed, and each could produce a variety of transcripts (Fig. 2c).

To ensure that the sequenced isoforms could be amplified, we selected three multiply matched coding region sequences and amplified the PacBio isoforms by RT-PCR in the three varieties ‘Rainer’, ‘Lapins’, and ‘21-21’. Comparison of these sequences with different matched sequences revealed SNPs or indels in some cases, indicating that they might be allelic genes with different alleles or homologous genes at different loci. For example, for the randomly selected gene *Pav_co4044289.1*, which is considered to be a defensin-like protein 1 gene, two different isoforms, c5390/f1p135/507 and c5422/f1p124/475, with 67.12% sequence similarity were found in each kind of pollen tube. c5390/f1p135/507 was located on chromosome 1 (as *Pav_co4044289.1*), and c5422/f1p124/475 could not be located on any of chromosomes 1 to 8. Intriguingly, a 113 bp coding sequence was inserted in both of the isoforms, and the two isoforms could be amplified from all three varieties, showing a good translation ability, indicating that the encoding ORF of *Pav_co4044289.1* was longer in the pollen tube than in the genome (Fig. 2d). In addition, two isoforms, c14252/f1p1/1541 and c17161/f1p1/1367, were found to match *Pav_sc0000044.1*, a gene associated with transcription initiation (Fig. 2e). The three sequences were all detected in pollen tubes from each of the three cultivars, and the two newly identified isoforms that shared the highest sequence similarity were located at the same position on chromosome 7, indicating that they may represent homologous genes with the same alleles or different isoforms from the same locus. The last selected gene was *Pav_sc0000103.1*, an E3-ubiquitin protein ligase RING1-like gene. One indel and two SNPs were detected among the two isoforms in all three cultivars of sweet cherry tested (Fig. 2f). Together, these results indicated that single-molecular long-read sequencing could be useful for identifying mRNAs and clustering homologs.

### GO and KEGG analysis of pollen tube transcripts

To determine the functions of the different transcripts in sweet cherry pollen tubes, we annotated the long-read sequencing results by using NCBI BLASTn (https://blast.ncbi.nlm.nih.gov/), where 19.1% (3031) of the transcripts could be annotated as peach (*Prunus persica*) transcripts, and 74.9% (11861) of genes could be annotated as plum (*Prunus mune*) genes, suggesting a close relationship among these genes in Rosaceae. To analyze the isoforms based on their functions using the Gene Ontology (GO) annotations, we first annotated them at the eggNOR database (http://eggnogdb.embl.de/#/app/home)^34^. Then, 15,836 annotated isoforms were annotated according to GO terms and classified by WEGO (http://wego.genomics.org.cn/)^35^. A total of 10,791 isoforms (69.1%) were annotated by WEGO, and 7300, 9791, and 3285 isoforms were annotated to the three categories of Biological Process, Molecular Function and Cellular Component (Supplemental Table 2).

**Fig. 3:**
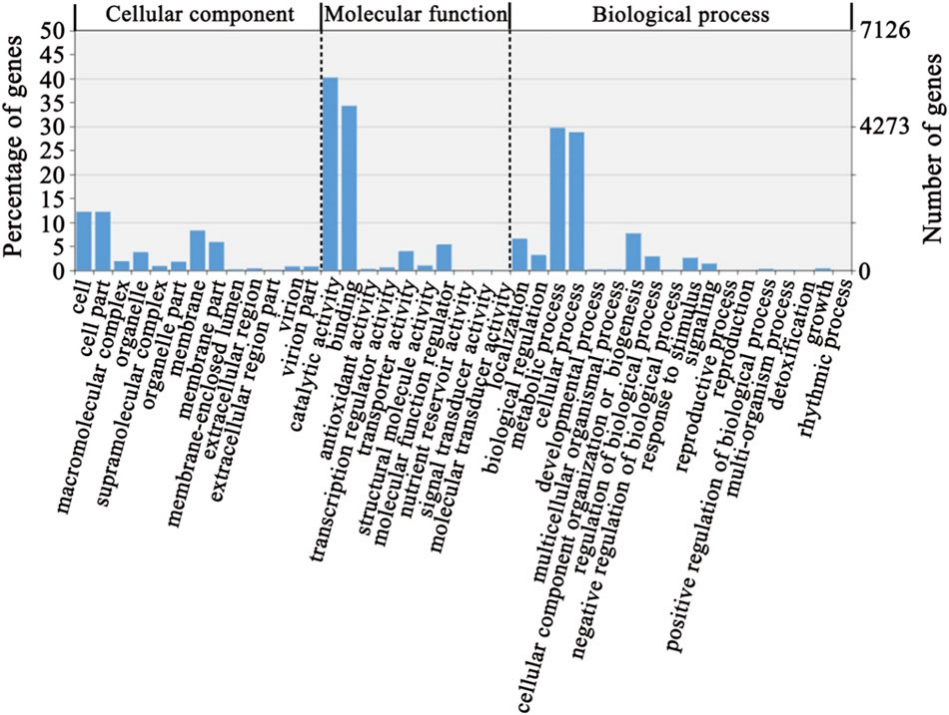
GO analysis of transcriptome results. The number of isoforms in each GO category classified by WEGO.

In the Cellular Component category, the annotated isoforms were classified into 13 categories (Fig. 3). The top three categories with the greatest numbers of genes were Cell, Cell Part and Membrane, which included 1936, 1936 and 1320 isoforms, respectively. In the Molecular Function category, annotated isoforms were classified into 10 categories (Fig. 3). The top three categories with the highest numbers of genes were Catalytic Activity, Binding and Molecular Function Regulator, which included 6365, 5432, and 850 isoforms, respectively. In the Biological Process category, the annotated isoforms were classified into 18 categories (Fig. 3). The top three categories with the highest numbers of genes were Metabolic Process, Cellular Process and Cellular Component Organization or Biogenesis, which included 4696, 4556, and 1219 isoforms, respectively.

**Fig. 4:**
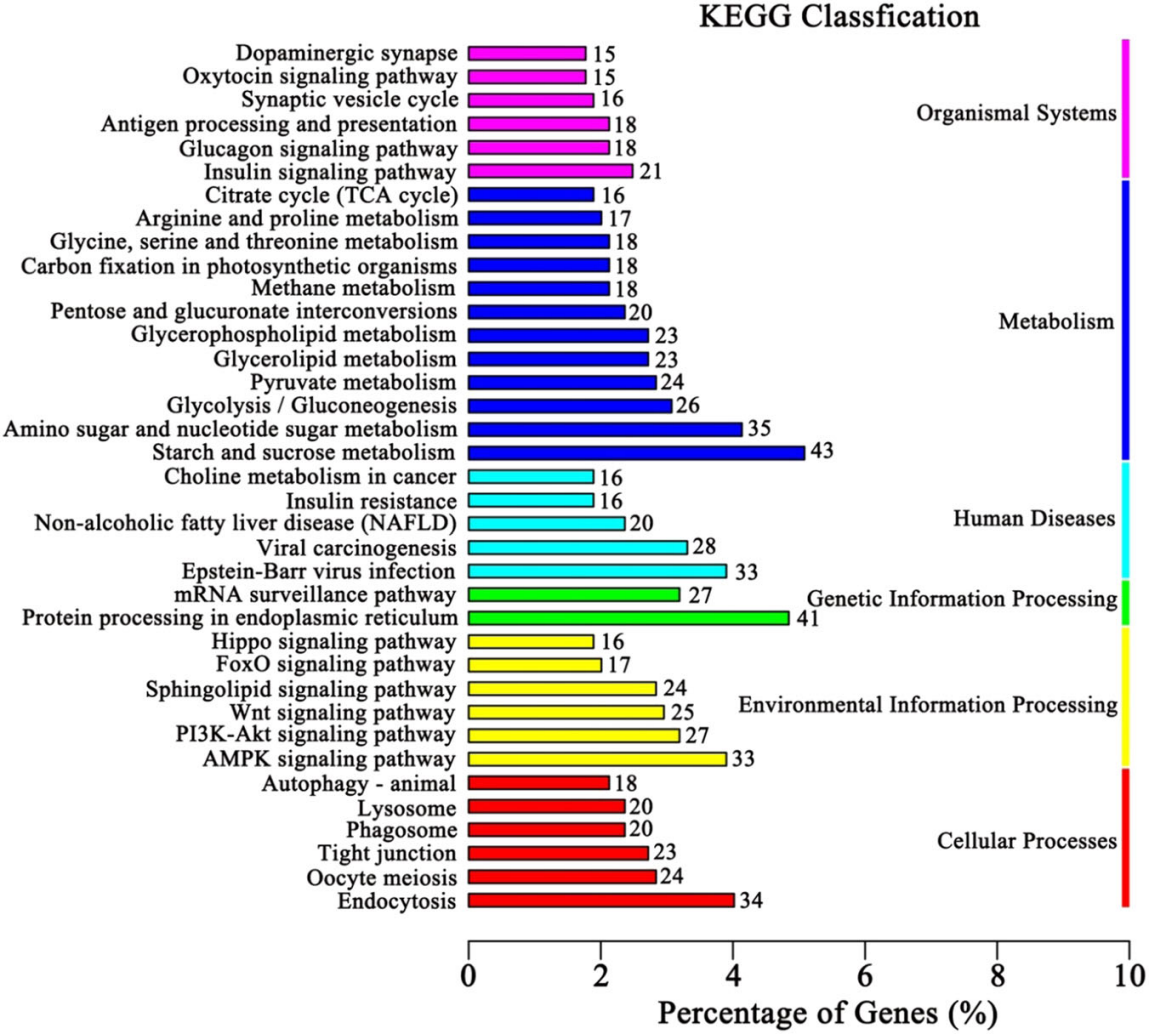
KEGG analysis of PacBio results using the KEGG database. Genes were enriched in different categories in the KEGG analysis. According to the enriched gene numbers, the top 40 categories belonging to six classes are shown. The *x*-axis represents the percentage of enriched genes among the background genes in different categories.

KEGG pathway analysis was also used to understand the genes expressed during pollen elongation. A total of 111 pathways, including 1517 genes, were enriched in the transcriptome of the pollen tubes. Among these pathways, the top 40 categories are shown in Fig. 4. The top five categories were Starch and Sucrose Metabolism (ko00500), Protein Processing in Endoplasmic Reticulum (ko04141), Amino Sugar and Nucleotide Metabolism (ko00520), Endocytosis (ko04144) and AMPK Signaling Pathway (ko04152), indicating that these pathways play important roles in pollen tube elongation. Among the six categories, as most of the isoforms were included in two categories: Metabolism (352 isoforms) and Environment Information Processing (286 isoforms), we focused on these two categories (Supplementary Fig. 4a, b). Twenty pathways in the Metabolism category, including Glycolysis/Gluconeogenesis (ko00010), Fructose and Mannose Metabolism (ko00051), Valine, Leucine and Isoleucine Degradation (ko00280), Sphingolipid Metabolism (ko00600), Starch and Sucrose Metabolism and Amino Sugar and Nucleotide Metabolism, and twenty pathways in the Environmental Information Processing category, including the PI3K-Akt Signaling Pathway (ko04151), the MAPK Signaling Pathway (ko04010) and the mTOR Signaling Pathway (ko04150), as well as the AMPK Signaling Pathway, were enriched (Supplementary Fig. 4a, b), indicating that these pathways also play important roles in pollen tube elongation.

### Analysis and identification of AS

**Fig. 5:**
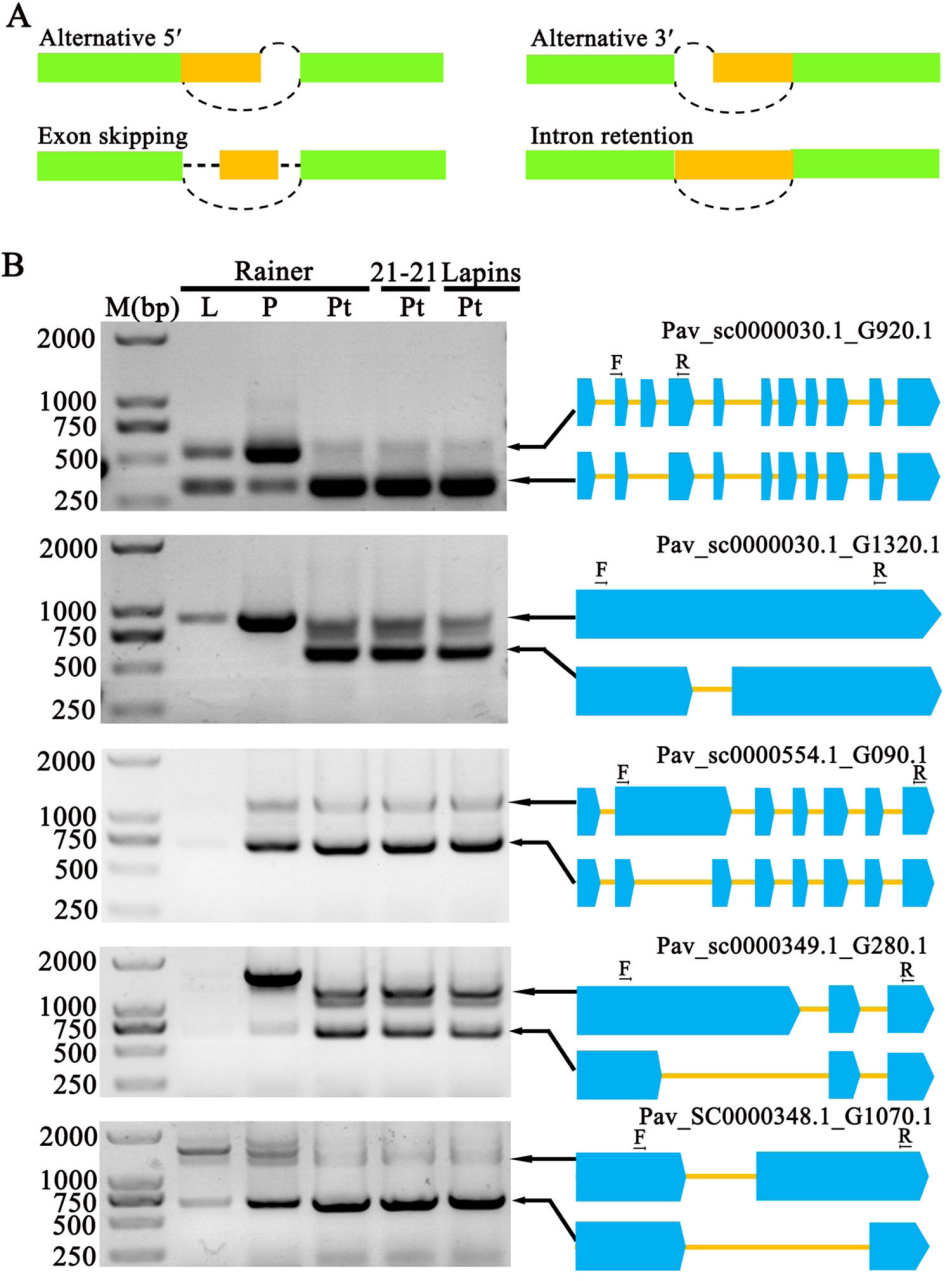
Analysis and validation of AS. **a** Schematic diagram of four different types of AS. **b** Four randomly selected genes: Pav_SC0000030.1_G920.1, Pav_SC0000030.1_G1320.1, Pav_SC0000554.1_G090.1 and Pav_SC0000349.1_G280.1 were amplified by RT-PCR, and images of an agarose gels containing the products are provided on the left. A schematic diagram of the AS of different genes is shown on the right, and the blue blocks represent exons in different genes. L leaf, P pistil, Pt pollen tube.

AS contributes to a variety of biological processes. Single-molecule long-read technology provides a more accurate and efficient way to identify AS events than earlier short-read sequencing approaches^36^. In our research, we first mapped the PacBio results to the genome of sweet cherry to produce a bam file. Then, we used SpliceGrapher software to predict four types of AS events: alternative 5′, alternative 3′, exon skipping, and intron retention^37^ (Fig. 5a). We identified a total of 2243 AS events in four groups. The intron retention group was the main group, comprising 37.58% of the AS events (Supplementary Table 3).

To validate the accuracy of the results, we selected seven random genes with different functions from various chromosomes (Table 2) and amplified them using specific primers (Supplementary Table 5). The results confirmed that all of the AS events in the seven genes occurred in the pollen tubes of all three varieties of sweet cherry (Fig. 5b, Supplementary Fig. 5). For example, the Pav_SC0000030.1_G920.1 gene, encoding a Beta-1, 3-galactosyltransferase, was located on chromosome 1. We found that an exon skipping event occurred in this gene, with the third exon being skipped. For the Pav_SC0000030.1_G1320.1 gene, which is located on chromosome 1, an intron retention event occurred. For the Pav_SC0000554.1_G090.1 and Pav_SC0000349.1_G280.1 genes, which are located on chromosome 2 and chromosome 6, an intron retention event and an alternative 5′ event were identified (Fig. 5b). The Pav_SC0000055.1_G560.1, Pav_SC0000087.1_G520.1 and Pav_SC0000348.1_G1070.1 genes were located on chromosomes 7, 6, and 7, and intron retention, alternative 5′ and alternative 3′ AS events were found in these genes (Fig. 5b, Supplementary Fig. 5). The fact that AS events occurred in different kinds of pollen tubes indicated that this is a general biological phenomenon in sweet cherry. It also showed that the proportion of splicing isoforms differs from gene to gene.

**Table 2:** Information for genes subjected to AS validation.

Gene ID	Gene function	Gene location	Interval of gene locus
Pav_SC0000030.1_G920.1	Beta-1,3-galactosyltransferase 7	Chr1	36523773-36527728
Pav_SC0000030.1_G1320.1	Flavonol synthase/flavanone 3-hydroxylase	Chr1	36291063-36294690
Pav_SC0000554.1_G090.1	guanylyl cyclase domain containing 1	Chr2	20567898-20572852
Pav_SC0000349.1_G280.1	FRIGIDA-like protein 3	Chr6	15783383-15786743
Pav_SC0000055.1_G560.1	Calcium uniporter protein	Chr7	9851745-9854819
Pav_SC0000087.1_G520.1	Insoluble beta-fructofuranosidase	Chr6	5021693-5025107
Pav_SC0000348.1_G1070.1	Actin-like protein	Chr7	12942653-12945173

AS is sometimes limited to specific tissues^36^. To determine whether there are also tissue-specific AS events in sweet cherry, we further detected the AS events of the seven genes in the pistil and leaf (Fig. 5b, Supplementary Fig. 5). We observed differences in AS among tissues. For example, the Pav_sc0000030.1_G920.1 gene showed a difference in splicing preference between the pistil and pollen tube. Other AS-affected genes, such as Pav_SC0000030.1_G1320.1, Pav_SC0000554.1_G090.1, Pav_SC0000349.1_G280.1, and Pav_SC0000055.1_G560.1, also showed tissue-specific AS. In particular, Pav_SC0000030.1_G1320.1, encoding a protein that participates in flavonol synthesis, underwent AS only in pollen tubes, indicating that its protein product may play a specific role in sweet cherry pollen tubes. For the Pav_SC0000348.1_G1070.1 gene, although an AS event occurred in the pistil, leaf, and pollen tube, the expression level of the isoforms produced from the gene differed among tissues.

### Analysis and identification lncRNAs

**Fig. 6:**
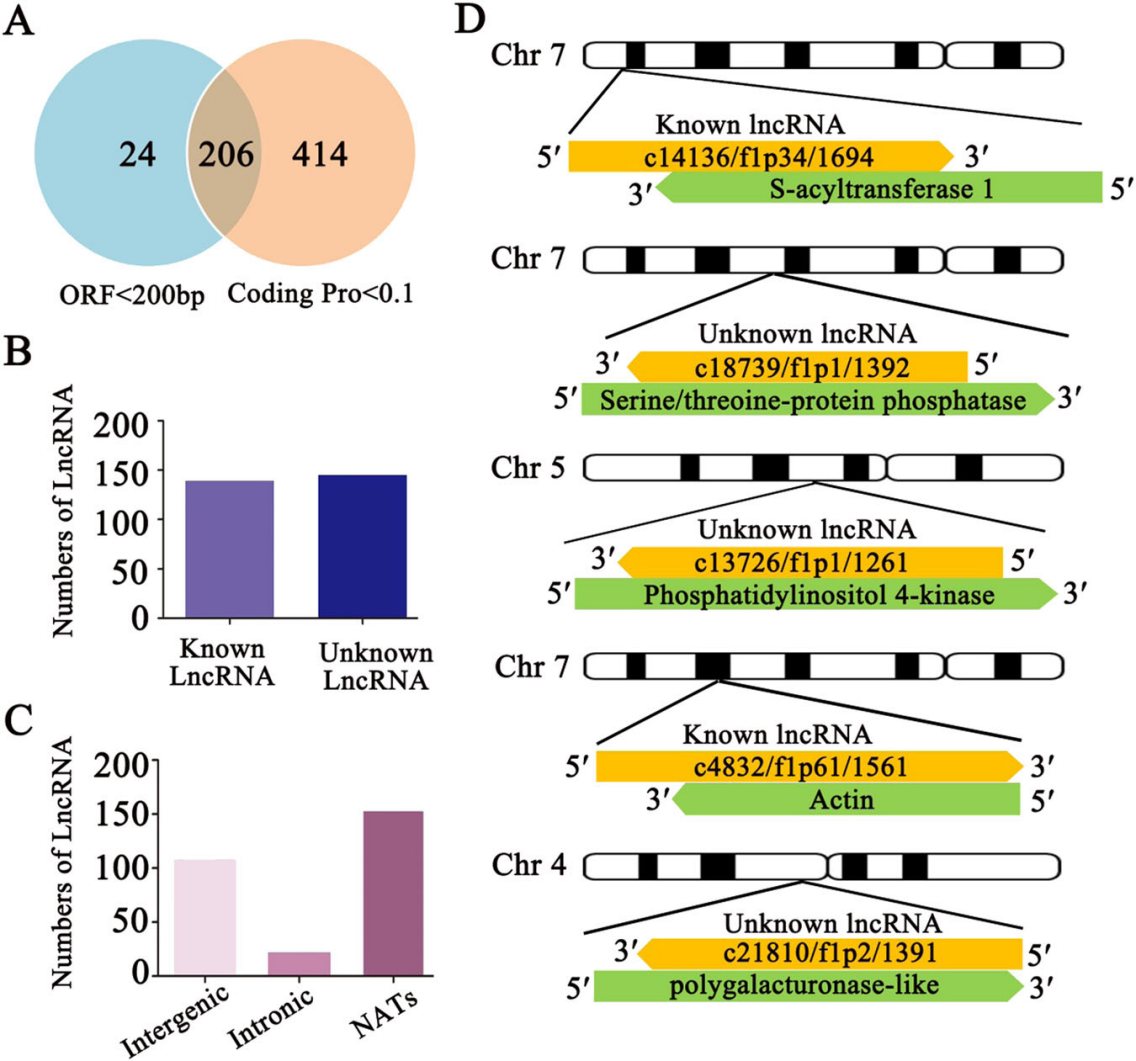
LncRNA prediction and statistics and the search for complementary genes. **a** LncRNA screening based on coding potential and ORF length. **b** Classification of known and unknown lncRNAs. **c** Classification of intronic, intergenic, and NAT-lncRNAs. **d** The genomic locations of five randomly selected lncRNAs and their complementary genes are indicated in a diagrammatic illustration.

The importance of LncRNAs has only recently been recognized, and they have not previously been studied in sweet cherry pollen tubes. Thus, we next screened probable lncRNA sequences in our data. As the length of lncRNAs should be more than 200 nt, we first observed the length of our transcripts, and we found that the length of the shortest transcript was 308 nt. Then, all the transcripts were subjected to BLAST searches against the NCBI-nt database to identify annotated lncRNAs, and a total of 130 lncRNAs were identified. To obtain more lncRNA signals from our data, we next removed the housekeeping lncRNA-like tRNA and rRNA sequences from our data and screened the candidate lncRNAs according to two criteria: an open reading frame (ORF) of less than 200 bp and a coding potential of less than 0.1. We first detected the coding potential of each isoform using CPAT software^38^. Among all the detected transcripts, most (13460) showed coding potential in the range of 0.9–1.0; 2118 of the sequences showed coding potential in the range of 0.1–0.9, and only 620 transcripts showed a coding potential of less than 0.1 (Supplementary Fig. 6a). Furthermore, we found that most transcripts contained a relatively long ORF (500–4500 bp), while 250 had very short ORFs of less than 200 bp (Supplementary Fig. 6b). Based on these findings, 206 transcripts from pollen tubes were considered to be candidate lncRNAs (Fig. 6a). Comparison with the lncRNAs identified via BLAST analysis revealed 154 newly identified lncRNAs. Thus, we identified 284 lncRNAs. Comparison with the NONCODE database (http://www.noncode.org/) showed that 139 were included in NONCODE, and 145 were not found; the latter group was considered to represent new lncRNAs unique to sweet cherry (Fig. 6b).

LncRNAs show diverse functional characteristics at the molecular level; one common model is that lncRNAs act as NATs that regulate the expression levels of complementary genes^21^. To investigate whether a similar process exists in sweet cherry pollen tubes, we next screened the complementary sequence of each lncRNA identified. We found that 154 of the lncRNAs from pollen tubes exhibited sequences that were complementary to genes with high coding potential, implying that they could act as NATs. The complementary genes with functional annotations are listed in Supplementary Table 4, and some of the complementary genes matched multiple NATs. The total number of complementary genes was 54, and the numbers of matches for each complementary gene are provided in Supplementary Table 4. In addition to NATs, lncRNAs can be located in intronic or intergenic locations. Hence, we performed an analysis to confirm the locations of the remaining lncRNAs. We found that 22 of the lncRNAs were located at intronic positions, and the other 108 were located at intergenic positions (Fig. 6c).

Next, we selected five NAT-lncRNAs and their complementary genes for subsequent analysis. Two of these NAT-lncRNAs, c14136/f1p34/1694, and c4832/f1p61/1561, were known, which were both sense sequences and were located at different positions on chromosome 7. Their complementary genes could encode the S-acyltransferase and actin proteins, respectively (Fig. 6d). The remaining three (c18739/f1p1/1392, c13726/f1p1/1261, and c21810/f1p2/1391) were newly identified NAT-lncRNAs, with antisense sequences on chromosomes 7, 5, and 4, respectively (Fig. 6d). Their complementary genes could encode serine/threonine-protein phosphatase, phosphatidylinositol 4-kinase and a polygalacturonase-like protein. The complementary sequences of the NAT-lncRNAs were identified as sequences encoding proteins with different functions. BLAST analysis using these NAT-lncRNAs and their complementary genes as queries against the sweet cherry genome showed that each of the lncRNAs and their complementary gene groups were located at only one position, which further indicated that the NAT-lncRNAs were transcribed in the reverse direction of the complementary genes and could be NAT-lncRNAs of each complementary mRNA (Fig. 6d).

### Functional identification of NAT-lncRNAs and their complementary genes in sweet cherry pollen

**Fig. 7:**
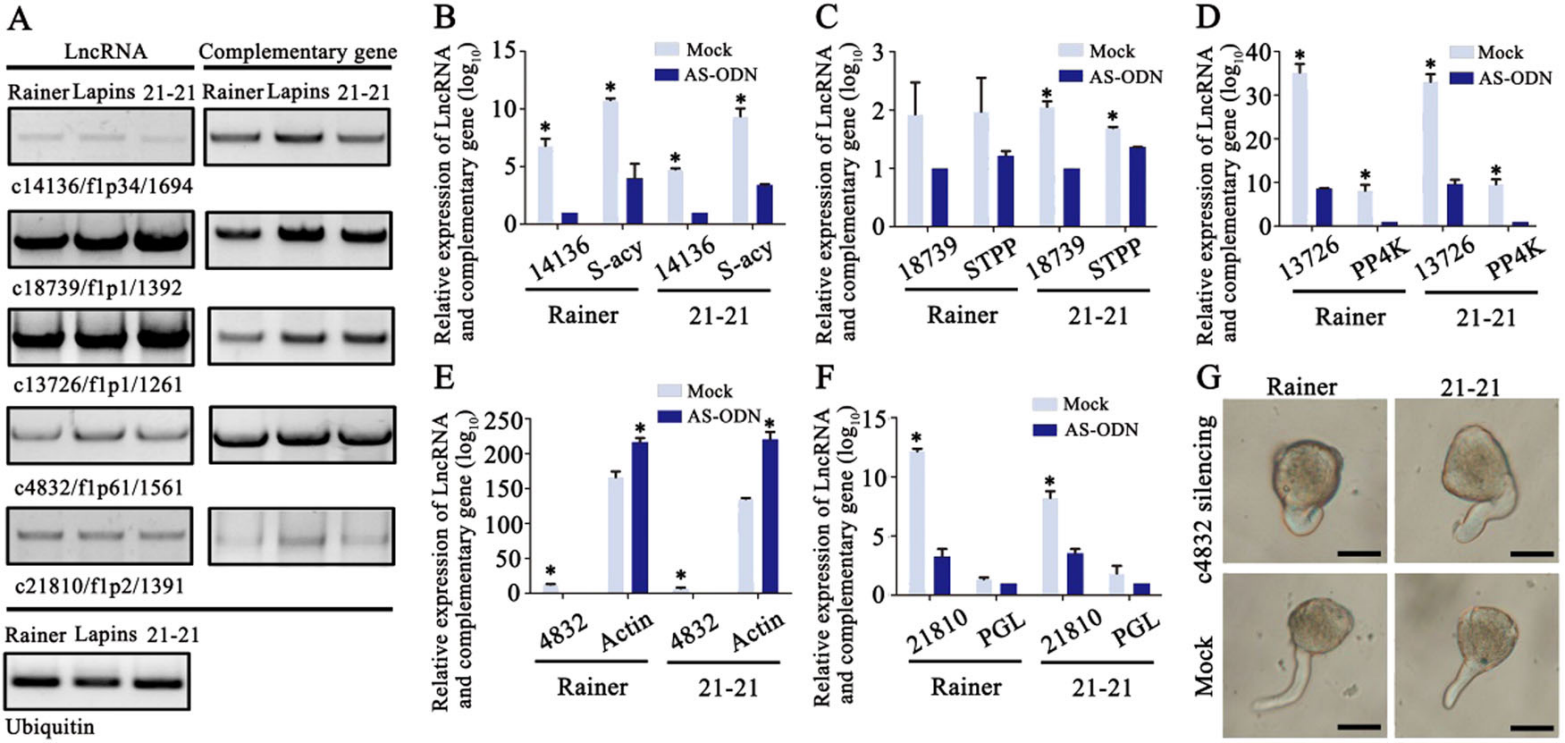
Cloning and qRT-PCR analysis of lncRNAs and their complementary genes. **a** RT-PCR of lncRNAs and their complementary genes in three varieties. **b**–**f** qRT-PCR analysis showing the expression levels of lncRNAs and their complementary genes in ‘Rainer’ and ‘21-21’ when the lncRNAs were suppressed by antisense transfection. *Student’s *t*-test: *P* < 0.05, bar: SD. 14136: c14136/f1p34/1694, S-acy S-acyltransferase 1, 18739: c18739/f1p1/1392, STPP: Serine/threonine-protein phosphatase, 13726: c13726/f1p1/1261, PP4K: phosphatidylinositol 4-kinase, 4832: c4832/f1p61/1561, 21810: c21810/f1p2/1391, PGL: polygalacturonase-like. **g** Growth condition of pollen tubes from different sweet cherry varieties when the lncRNA c4832/f1p61/1561 was silenced.

To validate the reliability of the high-throughput sequencing, we detected the sequences of the five NAT-lncRNAs and their complementary sequences by RT-PCR analysis (Fig. 7a). First, we extracted the RNA of pollen tubes from three different varieties of sweet cherry, ‘Rainer’, ‘Lapins’, and ‘21-21’, and then detected the five lncRNAs and their complementary sequences by RT-PCR analysis. All of the NAT-lncRNAs and their complementary gene transcripts were consistent with the sequencing results (Fig. 7a), indicating the high reliability of the analysis.

According to previous research, NAT-lncRNAs positively or negatively regulate the expression of their sense transcripts using diverse transcriptional or posttranscriptional mechanisms^39^. To explore the relationship between NAT-lncRNAs and their complementary gene transcripts, antisense transfection assays were performed to suppress the expression levels of the five NAT-lncRNAs and to then detect the expression of NAT-lncRNA complementary transcripts. For the NAT-lncRNAs of c14136/f1p34/1694, c18739/f1p1/1392, and c13726/f1p1/1261, when their expression levels were suppressed by antisense transfection, their complementary genes were downregulated to different degrees (Fig. 7b–d). However, when the NAT-lncRNA of c4832/f1p61/1561 were suppressed by antisense transfection, its complementary gene was upregulated (Fig. 7e). After suppressing the NAT-lncRNA of c21810/f1p2/1391 by antisense transfection, we found that the expression of the complementary transcript, a polygalacturonase-like gene, was not changed, which means that this NAT-lncRNA may not have the ability to regulate its complementary gene (Fig. 7f). These results suggest that different NATs may regulate their complementary genes in different ways.

As c4832/f1p61/1561 is complementary to an actin gene, we sought to observe the growth condition of pollen tubes in different sweet cherry varieties and detect whether the silencing of c4832/f1p61/1561 would affect pollen tube morphology or not. When the NAT-lncRNA c4832/f1p61/1561 was suppressed, 62.0% and 68.5% of the pollen tubes of ‘Rainer’ and ’21-21’ showed abnormal growth, respectively. However, in the mock treatment, only 3% and 5.7% of the pollen tubes of ‘Rainer’ and ’21-21’ showed abnormalities, respectively (Fig. 7g). These results suggest that c4832/f1p61/1561 may regulate the growth condition of pollen tubes via actin genes.

## Discussion

As sweet cherry is one of the most popular commercial fruit trees worldwide, large-scale molecular breeding of this species may be a useful means of generating new varieties and contributing to the sweet cherry industry. High-throughput sequencing of genomes and transcriptomes can produce large amounts of data, and the technology of long-read sequencing is helpful for whole-genome sequencing and cDNA analysis^5,7,40^; however, research using this technology has not previously been reported in sweet cherry.

In this study, we obtained full-length transcripts of sweet cherry pollen tubes using the PacBio RS II platform. In the isoform analysis, 3438 of 43,673 coding region sequences extracted from the genome of sweet cherry matched isoforms found in the PacBio results. Thus, we confirmed that these specific genes were expressed in pollen tubes. Furthermore, 129 of the identified isoforms did not match any transcribed sequence and therefore seemed to be novel. In long-transcript-sequencing-related studies in maize and cotton (*Gossypium hirsutum*)^5,36^, large numbers of novel transcripts were identified through comparison with reference genomes of maize and cotton, respectively. These results indicated that long-read sequencing is helpful for identifying novel isoforms or genes. Nevertheless, our approach enriched the data for nonredundant isoforms and provided a more complete pollen tube transcriptome. To further elucidate the functions of different transcripts in sweet cherry pollen tubes, our data were first annotated by using the WEGO database (Fig. 3), and 40 GO terms were observed to be enriched. We found that 6365 genes were enriched for the GO term of catalytic activity (GO: 0003824), which suggested that these genes play a very significant role during pollen tube growth. To better understand the mechanism of pollen tube elongation, we next annotated our data by using the KEGG database (Fig. 4). The pathways of the metabolism and environmental information processing categories were enriched in the transcriptome of sweet cherry pollen tubes. The pathways related to the metabolism of starch, sugars and amino acids and some signal transduction pathways were included in these enriched pathways, indicating that these pathways play important roles in pollen tube elongation. Little is known about AS in sweet cherry. Nearly 40% of the AS events identified involved intron retention, which is consistent with similar analyses in other plant species showing that intron retention accounts for a large percentage of AS events in plants^41,42^. After we identified seven AS events among pollen tube genes, we found that two of the genes may participate in ion exchange or pollen tube elongation. Pav_SC0000055.1_G560.1 and Pav_SC0000348.1_G1070.1 encode a calcium uniporter protein and an actin-like protein, respectively. Calcium ions are essential for pollen tube germination^43^, and calcium uniporter proteins function as part of the inner membrane transporters of Ca^2+ ^^44^. The observation that Pav_SC0000055.1_G560.1 is alternatively spliced suggests that it may be involved in the process of pollen tube growth through an unknown mechanism. The actin protein might form part of the cytoskeleton of the pollen tube and could be closely related to pollen tube elongation^45^. However, the function of the actin-like protein is not clear, and the observation of AS for this gene suggests that its function in pollen tube growth should be investigated further. Although we did not detect all the predicted AS events and did not elucidate the functions of AS in the sweet cherry pollen tube, we did confirm that AS occurs in sweet cherry and is sometimes tissue specific.

The identification and analysis of lncRNAs are key contributions of our study, which revealed tissue-specific expression and low expression levels compared with coding genes^5,21^. The observation that some lncRNAs and mRNAs exhibit the same structures, such as poly-A tails, transcriptional start sites (TSSs), and transcriptional termination sites (TTSs), makes it difficult to confirm lncRNA identity, leading to deficiencies in lncRNA prediction^46–50^. In this study, 284 lncRNAs were predicted, and we determined that 139 of these lncRNAs were novel. LncRNAs localize in either the cell nucleus or cytoplasm and regulate gene expression at the transcriptional or posttranscriptional level by binding to nucleotides such as complementary RNA, homologous DNA, and miRNAs or to proteins such as histones or transcription factors^49^.

The process whereby lncRNAs bind to proteins is difficult to predict—requiring a biochemical approach such as RNA immunoprecipitation (RIP) or photoactivatable ribonucleoside-enhanced crosslinking and immunoprecipitation (PAR-CLIP) assays^51^, and the screening of lncRNAs for the ability to function as natural antisense transcript sequences (NATs) complementary to mRNAs could be made much easier by bioinformatic analysis. Therefore, we screened for NAT function, and we successfully identified a considerable number of lncRNAs that act as NATs (154). The functions of the complementary genes of the NATs was analyzed, and ubiquitin-related genes such as the 26S proteasome non-ATPase regulatory subunit 10, E3 ubiquitin-protein ligase RHF2A-like, tubby-like F-box protein 5 and probable E3 ubiquitin-protein ligase ARI2 were identified, which indicated that NAT-lncRNAs may regulate the ubiquitin system during pollen tube growth (Supplementary Table 4). We also identified genes such as S-acyltransferase 1, phosphatidylinositol 4-kinase, polygalacturonase-like, and serine/threonine-protein phosphatase, which suggested that NATs may participate in basic physiological reactions during pollen tube elongation (Supplementary Table 4). These results suggested a common pattern of NAT-lncRNA function in pollen. Although we found some relationship between NAT-lncRNAs and mRNAs that encode proteins, further analysis is needed to confirm whether the NAT-lncRNAs truly regulate gene expression or affect biological processes in pollen tubes. Thus, an antisense transfection assay was used for further investigation, and it was shown that the NAT-lncRNAs could in fact regulate their complementary coding genes. Furthermore, we found that one NAT-lncRNA may participate in the regulation of the actin gene to affect the growth conditions of pollen tubes, which suggests that NAT-lncRNAs may affect some RNA-based processes when sweet cherry pollen tubes are growing. Due to technical limitations, the recently identified nonpolyadenylated class of NAT-lncRNAs could not be detected among our results. Nevertheless, our research provides useful insights into the pollen tube transcriptome in sweet cherry; the comprehensive information provided here should enhance our understanding of male gametogenesis in sweet cherry and could serve as a reference for future studies of sweet cherry pollen tubes.

## Materials and methods

### Plant materials

The sweet cherry cultivars ‘Rainer’, ‘Lapins’, and ‘21-21’ were grown at the Beijing Academy of Agriculture and Forestry. ‘21-21’ is an offspring of self-pollinated ‘Lapins’. The anthers of each variety were collected when the flowers had just begun to open. Then, the anthers were carefully separated from the filaments and dehydrated until the pollen grains were released.

### Sample preparation and RNA extraction

The pollen grains of each variety were suspended in liquid germination medium containing 10% (w/v) sucrose, 0.01% (w/v) H_3_BO_3_, and 0.015% (w/v) CaCl_2_. After 3 h of culture at room temperature, the pollen germination rate was determined. Then, the liquid germination medium was removed using a pipette, and the pollen was washed three times with PBS buffer. The leaves and pistils of each variety were also sampled in the bloom period and stored at −80 °C. RNA was extracted from the pollen, leaf, and pistil samples using an EASY Spin Plus Plant RNA Kit (RN40, Aidlab Company, Beijing, China) and stored at −80 °C.

### Library preparation and PacBio sequencing

The RINs (RNA Integrity Numbers) of the ‘Rainier’, ‘Lapins’ and ‘21-21’ pollen tube RNAs were 9.2, 9.0, and 9.3, respectively. One microgram of Pollen tube RNA from each variety was mixed as the sample for library construction. The RNA samples were reverse transcribed using the Clontech SMARTer cDNA synthesis kit (TaKaRa Company) in separate PCR tubes. To generate barcoded FL cDNAs, three RT reactions were run in parallel. PCR optimization was performed to determine the optimal amplification cycle number for the downstream large-scale PCR assays. A single primer (primer IIA from the Clontech SMARTer kit: 5′-AAG CAG TGG TAT CAA CGC AGA GTA C-3′) was used for all amplification reactions following RT. The large-scale PCR products were purified with AMPure PB beads, and quality control (QC) was performed on a 2100 BioAnalyzer (Agilent). The different size fractions eluted from the run were subjected to QC and pooled in equimolar ratios for subsequent reamplification to yield three libraries (1–2, 2–3, 3–6). A total of 6 SMRT cells (Supplementary Table 1) were sequenced on the PacBio RS II platform using P6-C4 chemistry with 3–4 h movies at the Tianjin Biological Chip Technology Company.

SMRT analysis software (version: smrtanalysis_2.3.0.140936.p4.150482) was used to identify the final isoforms from the raw reads. First, the raw reads were processed into error-corrected reads of inserts with minFullPass = 0 and minPredictedAccuracy = 0.80. Then, the full-length, nonchimerric (FLNC) transcripts were determined by searching for the polyA tail signal and the 5′ and 3′ cDNA primers. Iterative clustering for error correction (ICE) was used to obtain consensus isoforms. Nonfull-length sequences were used to filter the consensus isoform sequences with Quiver software to obtain the polished isoforms, and the parameter was set at 0.99. The full-length and nonfull-length reads were aligned to the sweet cherry genome using the Genome Mapping and Alignment Program (GMAP)^52^. Next, indels and mismatches were corrected using the reference sweet cherry genome.

### Classification of isoforms, isoform annotation, and prediction of AS

The coding region sequence file for the sweet cherry genome was extracted with a Python script, and sequence alignment between the coding sequences and isoforms from our sequencing data was then performed by using ClustalW software (https://www.genome.jp/tools-bin/clustalw)^53^ with a cut-off e-value of 1e-10. To validate the results of the alignment, genomic DNA was isolated from fresh leaves of ‘Rainer’, ‘Lapins’, and ’21-21’ by using the EZ Spin Column Genomic DNA Isolation kit (Biomega Inc., Foster City, CA) as described previously^54^. The pollen tube RNAs of the three varieties were also prepared, and cDNAs were produced with an OligodT primer. The six selected PacBio isoforms were compared with their matched coding region sequences by using DNAMAN software (version 7.0.2.176). Specific primers for c5390/f1p135/507 and c5422/f1p124/475 were designed to amplify their whole isoforms. Conserved primers were designed to amplify the isoforms of c14252/f1p1/1541 and c17161/f1p1/1367 and the isoforms of c13658/f2p1/1544 and c13933/f1p1/1499. PCR experiments at the DNA and RNA levels were performed, and the products were sequenced by Sanger sequencing to detect sequence variation, such as SNP and indels. The primers are listed in Supplementary Table 5.

For isoform annotation, the isoforms were first annotated by NCBI BLASTn (https://blast.ncbi.nlm.nih.gov/)^55,56^ with a cut-off e-value of 1e-10. For the GO and KEGG enrichment analyses, the isoform data were first annotated at the eggNOG eukaryotic database (version: 1.0.3), and WEGO 2.0 (http://wego.genomics.org.cn/) software was then used to enrich genes in different GO categories. The KEGG (https://www.kegg.jp) database was used to enrich genes in different KEGG terms. The KEGG annotation of the sweet cherry genome was used for the background KEGG terms.

For AS analysis, the PacBio results were first mapped onto the sweet cherry genome with GMAP software to produce bam files, and the analysis of AS was then performed with SpliceGrapher software (http://splicegrapher.sourceforge.net/)^37^. The analysis required both bam files after mapping and coding region sequence files from our data. The cDNAs of the pollen tubes of the three investigated cultivars were also used for validation. Specific primers for each AS event were designed to cover the splicing site. The primers are listed in Supplementary Table 5.

### Predication and qRT-PCR validation of LncRNAs and NATs

The length of each isoform was calculated with a Python script, and NCBI BLASTn (https://blast.ncbi.nlm.nih.gov/) was used to identify the annotated lncRNAs with a cut-off e-value of 1e-10. Housekeeping ncRNAs such as rRNAs and tRNAs were removed from the remaining isoforms, and coding potential was detected with Coding-Potential Assessment Tool (CPAT) software. The length of ORF in each isoform was also detected by using ORF software (http://embossgui.sourceforge.net/demo/getorf.html)^57^. Then, the candidates were selected. The final candidate lncRNAs were compared with the previous dataset (http://www.noncode.org/) and mapped on the genome (https://www.rosaceae.org/). To test the NATs in the obtained lncRNAs, the complementary sequences of the lncRNAs were first obtained with a Python script, and ClustalW software was then used to align the obtained sequences and transcripts after the housekeeping ncRNAs were removed, with a cut-off e-value of 1e-10.

To validate the prediction of lncRNAs and NATs, five randomly selected lncRNAs and their complementary coding mRNAs were detected. Total RNA from the pollen tubes of different varieties of sweet cherry was extracted by using the EASY spin Plus Plant RNA Kit (RN40, Aidlab Company, Beijing, China). Specific primers were designed to reverse transcribe the lncRNAs and their complementary sequences in different reactions to synthesize the first cDNA chain. Then, PCR experiments were performed with specific primers, which are listed in Supplemental Table 5.

### Antisense oligo experiments

Antisense oligonucleotide experiments were performed essentially as previously described^58^. A phosphorothioated antisense oligodeoxynucleotide (as-ODN) was synthesized (Sangon Biotech Co., Ltd., Beijing, China) to downregulate gene expression. For transfection, 20 μl (10 pmol) of oligonucleotides (as-ODN), 28 μl cytofectin buffer and 4 μl cytofectin were premixed and immediately added to 200 μl germination medium. Two hours later, pollen tubes were collected for RNA extraction. The same mixture without the oligonucleotides was used as a control. The antisense oligodeoxynucleotides for the antisense oligonucleotide experiments are listed in Supplementary Table 5.

### Real-time PCR assay of LncRNA and NATs

Total pollen tube RNA was extracted from different varieties of sweet cherry by using the EASY spin Plus Plant RNA Kit (RN40, Aidlab Company, Beijing, China). The first cDNA chain was synthesized with oligo-dT primers and different specific primers with the same content of RNA. Real-time PCR was performed using SuperReal PreMix Plus (SYBR green) (TianGen Biotech Company) and the conditions outlined above. The relative RNA abundance was calculated using the 2−ΔΔCT method, and the ubiquitin gene of sweet cherry was regarded as the reference gene. The primers for the real-time PCR assay are listed in Supplementary Table 5.

## Supplementary information

New version of Supplemental figures and tables
