# Supplementary material for: Functional identification of lncRNAs in sweet cherry (Prunus avium) pollen tubes via transcriptome analysis using single-molecule long-read sequencing
Source: Hortic Res. 2019 Dec 1;6:135. doi: 10.1038/s41438-019-0218-3 (PMC6885045; doi:10.1038/s41438-019-0218-3)
Supplement: Supplementary file 1 — New version of Supplemental figures and tables [file 41438_2019_218_MOESM1_ESM.pdf]

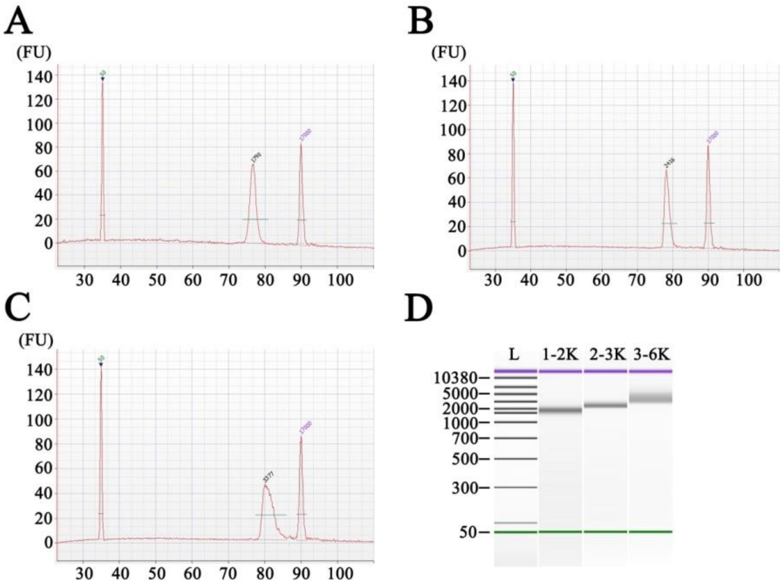

Supplemental Figure 1: Quality control of PacBio library in different size ranges. (A) Detection of nucleotide content in 1–2 kb library. (b) Detection of nucleotide content in 2–3 kb library. (c) Detection of nucleotide content in 3–6 kb library. (d) Gel photo of three libraries of different size by Bioanalyzer chip.

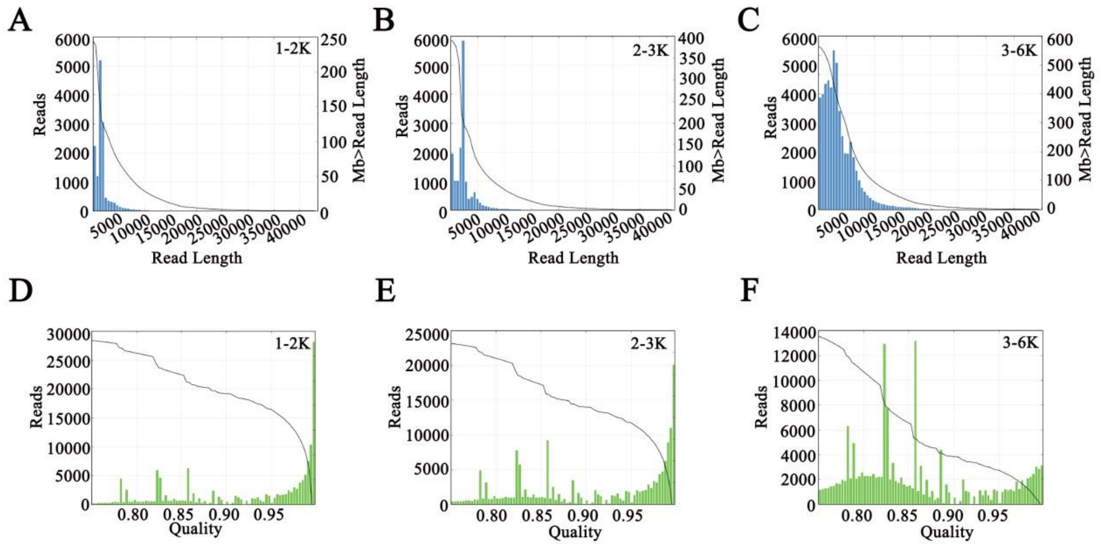

Supplemental Figure 2: Read length and quality statistics. (a) Distributions of read lengths in the 1–2 kb library. (b) Distributions of read lengths in the 2–3 kb library. (c) Distributions of read lengths in the 3–6 kb library. (d) Distributions of read qualities in the 1–2 kb library. (e) Distributions of read qualities in the 2–3 kb library. (f) Distributions of read qualities in the 3–6 kb library.

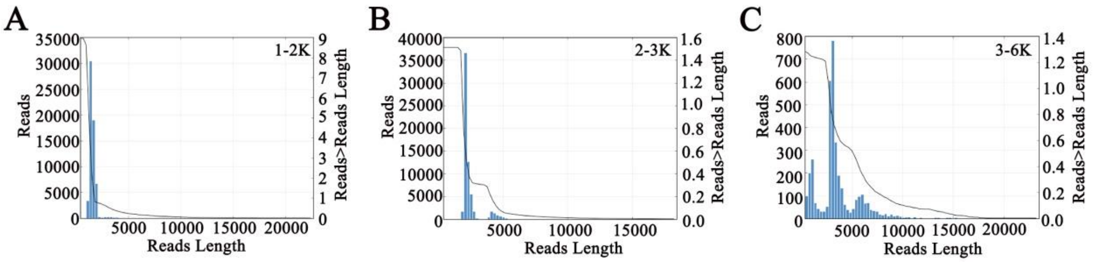

Supplemental Figure 3: Statistics for non-chimeric full-length reads. (a) Length distribution of full-length non-chimeric reads in the 1–2 kb library. (b) Length distribution of full-length non-chimeric reads in the 2–3 kb library. (c) Length distribution of full-length non-chimeric reads in the 3–6 kb library.

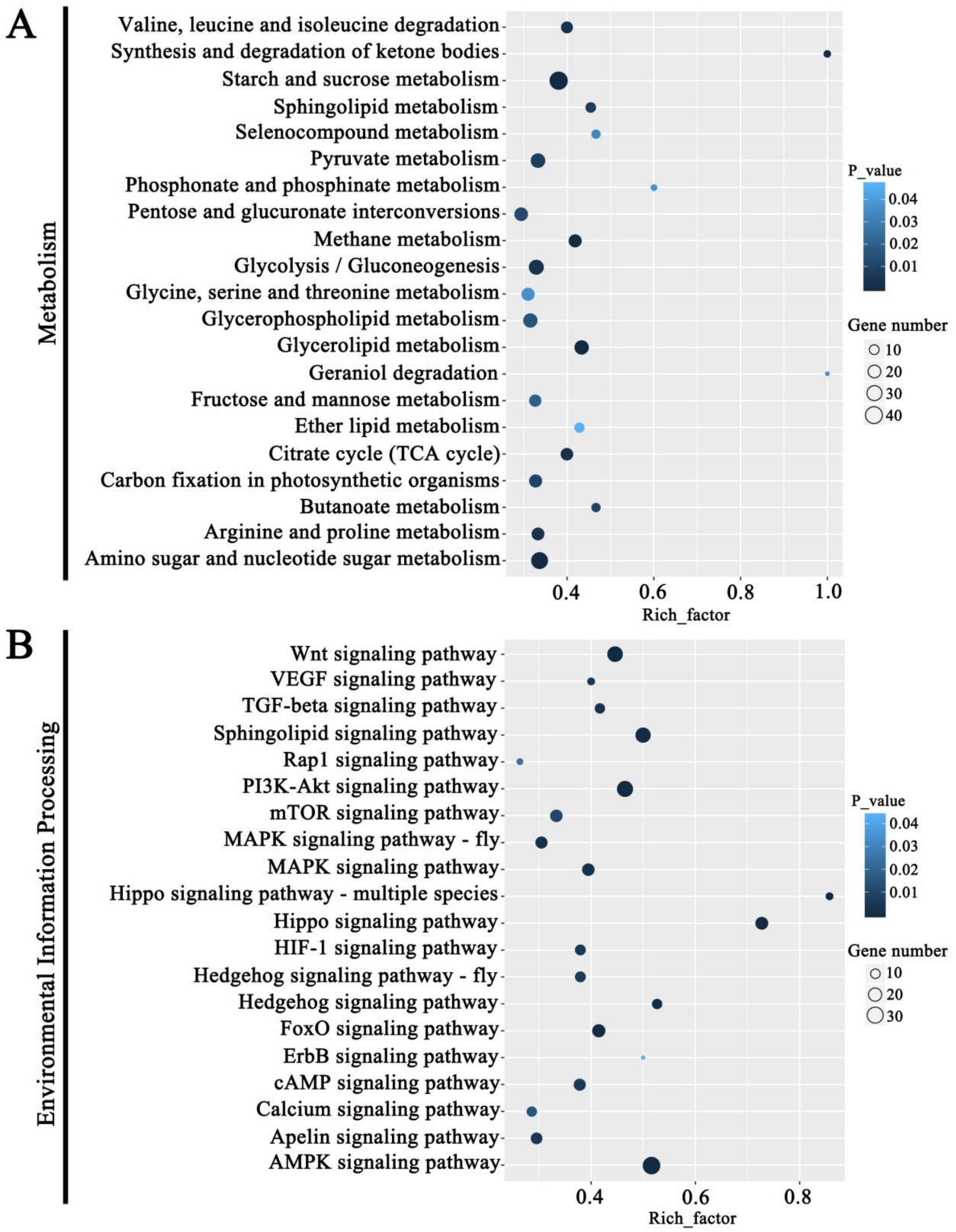

Supplemental Figure 4: KEGG analysis of the class metabolism and environmental information processing. (a) 21 KEGG terms were included in the class of metabolism. (b) 20 KEGG terms were included in the class of environmental information processing. The size of circle represents gene number. The depth of color represents the P-value. The x-axis represents the percentage of enriched genes to background genes in different categories.

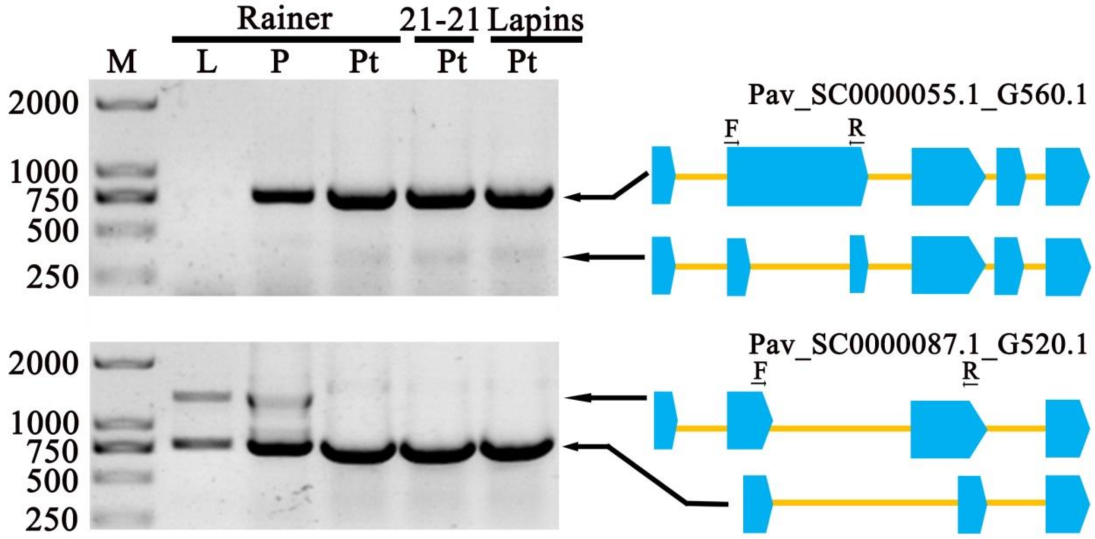

Supplementary Figure 5: Analysis of alternative splicing and validation. RT-PCR analysis of randomly selected genes and validation of alternative splicing. L: leaf, P: pistil, Pt: pollen tube.

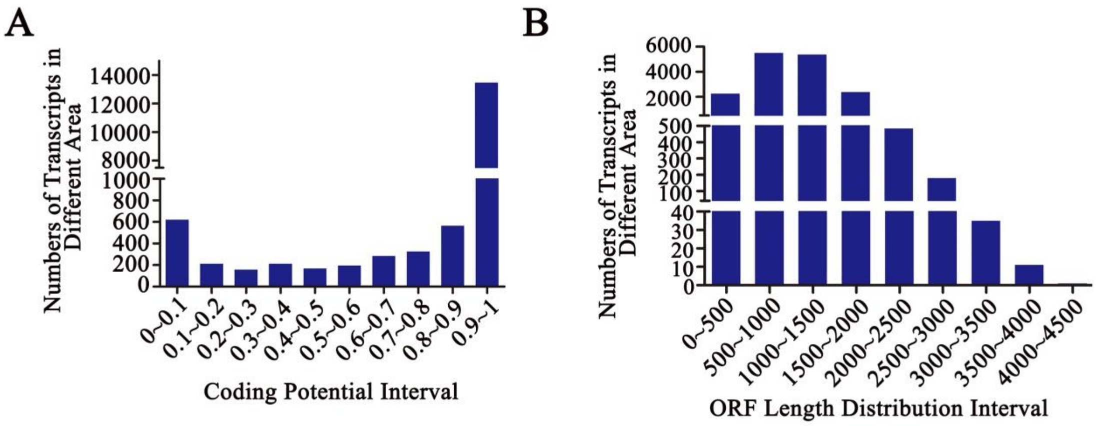

Supplementary Figure 6: Calculation of coding potential and ORF length statistics for every isoform. (a) Distribution of transcripts according to coding potential. (b) Distribution of transcripts according to ORF length.

Supplementary Table 1 Classification of varieties of reads from PacBio sequencing

Category	1—2 kb	2—3 kb	3—6 kb
Number of filtered short reads	16053	13945	7152
Number of full-length non-chimeric reads	61857	65467	3731
Number of five prime reads	77855	84905	48060
Number of three prime reads	84443	92847	54597
Number of full-length reads	62915	67564	30555
Average full-length non-chimeric read length	1592	2427	3594
Number of poly-A reads	81817	87870	42104
Number of non-full-length reads	64484	81499	121440

Supplementary Table 2 GO analysis of all isoforms

Category		Go analysis
Gene		15836
Annotated Genes		10791
	Biological	7300
Go Terms	Cellular	3285
	Function	9791
	Total	20376

Supplementary Table 3 Statistics for the different types of alternative splicing

	Alternative 5′	Alternative 3′	Exon skipping	Intron Retention	total
Events	358	298	744	843	2243

Supplementary Table 4 Complementary genes of the NAT-lncRNA with Functional annotations

No.	Functional annotations of complementary genes	Matched No.
1	26S proteasome non-ATPase regulatory subunit 10	1
2	S-acyltransferase 1	1
3	phosphatidylinositol 4-kinase	1
4	hypothetical protein (PRUPE_ppa005297mg)	1
5	actin	1
6	polygalacturonase-like	1
7	flagellar attachment zone protein 1	1
8	receptor-like cytosolic serine/threonine-protein kinase RBK1	1
9	transmembrane protein 53	1
10	disulfide-isomerase	1
11	tubby-like F-box protein 5	1
12	serine/threonine-protein phosphatase	1
13	putative SNAP25 homologous protein	1
14	translocation protein	1
15	probable galacturonosyltransferase-like 4	1
16	TMV resistance protein N-like	1
17	2-aminoethanethiol dioxygenase	1
18	protein KTI12 homolog	1
19	succinyl-CoA ligase [ADP-forming] subunit beta	2
20	pollen-specific protein SF3	2
21	heterogeneous nuclear ribonucleoprotein 1-lik	2
22	hypothetical protein (PRUPE_ppa010437mg)	2
23	DNA-directed RNA polymerase III subunit	2
24	mitochondrial pyruvate carrier 2-like	2
25	probable beta-1,3-galactosyltransferase	2
26	protein IQ-DOMAIN 1	2
27	pathogenesis-related protein PRMS-like	2
28	heterogeneous nuclear ribonucleo protein F	2
29	PRA1 family protein B1	2
30	fructose-bisphosphate aldolase cytoplasmic isozyme-like	3
31	protein CURVATURE THYLAKOID 1B	3
32	hypothetical protein (PRUPE_ppa027032mg)	3
33	hypothetical protein (PRUPE_ppa004811mg)	3
34	hypothetical protein (PRUPE_ppa008594mg)	3
35	hypothetical protein (PRUPE_ppa010002mg)	3
36	hypothetical protein (PRUPE_ppa003794mg)	3
37	transcription factor RF2b-like	3
38	hypothetical protein (PRUPE_ppa007029mg)	4
39	hypothetical protein (PRUPE_ppa022052mg)	4
40	hypothetical protein (PRUPE_ppa023498mg)	4
41	dnaJ homolog subfamily A member 2-like	4
42	pPn31C7 hypothetical proteins	4

43	hypothetical protein (PRUPE_ppa010711mg)	4
44	hypothetical protein (PRUPE_ppa017239mg)	5
45	shaggy-related protein kinase theta-like	5
46	hypothetical protein (PRUPE_ppa017163mg)	5
47	RNA polymerase beta subunit	5
48	hypothetical protein (PRUPE_ppa009725mg)	5
49	hypothetical protein (PRUPE_ppa024468mg)	5
50	phosphatidylinositol/phosphatidylcholine transfer protein SFH12-like	6
51	hypothetical protein (PRUPE_ppa013778mg)	6
52	pheophytinase	7
53	E3 ubiquitin-protein ligase RHF2A-like	8
54	probable E3 ubiquitin-protein ligase ARI2	9

Supplementary Table 5 Primers used in this study

For isoform classification		
Name	Sense primer	Anti-sense primer
c5390/f1p135/507	GATCTCTACTGCATATACACAAGCACAC	GATAGCAAAAGCAATTAACCCAGCAC
c5422/f1p124/475	GAGATCTCTACTTCATACACAAGCAGC	GCACAATTAA CCCAGCATAC AATACAG
c14252/f1p1/1541	GGTGGTTCTGCACAAACGTCACAG	CAACAAAGGCTTCTGAAGGATCAGG
c17161/f1p1/1367		
c13658/f2p1/1544	CTCAGCTTTACTTTTGCCACCAGTG	CGAATGGTATATGTGCTTACAGGGC
c13933/f1p1/1499		
For alternative splicing validation		
Name	Sense primer	Anti-sense primer
Pav_SC0000030.1_G920.1	GTAGCACCTGAATCCAATGGTCAG	CTCGCCTCTT TCTACTACTA AAAGC
Pav_SC0000030.1_G1320.1	GAATATGAAGCCATACCCATAGGG	TTTAGGTGGATTGTCCTCCTTCAC
Pav_SC0000554.1_G090.1	TGGTGAGAAACCATAGCTGAATTGG	CATCTGTAGCAGAGTCATACCCAC
Pav_SC0000349.1_G280.1	TGCTCACAAGGATACGTCTGAGGG	CTTGTTGGCG TTTACCAAAT TCACC
Pav_SC0000055.1_G560.1	TCAGTGTGGGGAAACATTGTGTGG	TGTATCAGAC TCATTAGCCC AACC
Pav_SC0000087.1_G520.1	AACTTCGTGTGGCTCCAGAAGAAC	AGGATGGATC CACCAATCCA GACAC
Pav_SC0000348.1_G1070.1	GAGAAGCTCAGGTTCATGGCCATTG	CGCTTCTGCTTCGCGTGAAACCTG
For identification of lncRNAs and complementary genes (anti-sense primer was used to reverse RNA to cDNA)		
Name	Sense primer	Anti-sense primer
c14136/f1p34/1694	GCAGAATAATGAATTAATTTCATTTTC	ATTCGCTTACCCATCAGAAAAATGGTCTC
S-acyltransferase 1	CGTATCAGTTATTCTCATAGTATATTG	AACGAAACGTAAAGCCTGTGTTGCATGG
c18739/f1p1/1392	GAATCTGGGGTTAGGGTTATCCTAATCC	GTGCAAACATCCACTAATAATAAAGTTCC
Serine/threonine phospatase	CTCCGTCCTCTCACCTCTGCTTTAAGC	AAAGTTCCATGCACCCTTGAGAGCC
c13726/f1p1/1261	GGGTTCCAGTTGTAACCTGGGAGTC	GACATAAATGAAGGGAAACTTCAGGC
Phosphatidylinositol 4-kinase	GTTGAAATCTTTGTGGGAGCTTCAAG	AACCTCTAAAACCTAGGTCACCAGGG
C4832/f1p61/1561	GAAGGGATTTCTGATTTCAACCAAACC	CTCAGCCATTCTGTTAAGACAGAGGC
Actin-like	GCCATTCTGTTAAGACAGAGGCAGAC	TTTGCTCGCCTCCATGTTCATGTCCC
c21810/f1p2/1391	GGTAGCGATATGTGCCATCAAAGCC	CTCTATCTTGGATGTCAAGATGGCG
Polygalacturonase-like	CTCAGGAGATGGCATTTTTGATGGCC	CCCGACGAATACTAGTACGTGATTAG
For Real-Time PCR of lncRNAs and complementary genes		
Name	Sense primer	Anti-sense primer
c14136/f1p34/1694	GACAAAGACATATACGCACAGGAAG	CACTTGTTTGCTATATCGTCCACCG
S-acyltransferase 1		
c18739/f1p1/1392	GATCGTGTTCAAGAGGTTCCACATG	CCATAACCAACTGATGAGCTCTTGC
Serine/threonine phospatase		
c13726/f1p1/1261	GGTTTGCAGAAGGGCAATCAACC	ATGGGGATTGTTCACTGCATTAGG
Phosphatidylinositol 4-kinase		
C4832/f1p61/1561	GGATGCCAAAATGGAGCCTCCAATCC	TTAGTGGAGGCTCCACTATGTTCCC
Actin		
c21810/f1p2/1391	CGCAGGTTAACAACTTGCTCATCTC	CGACACTATTGTTCGATGTTATGCC
Polygalacturonase-like		
For antisense transfection assay		

Name	phosphorothioated antisense oligodeoxynucleotide (* indicates the modified bases)
c14136/f1p34/1694 AS	G*G*G*TGGGTCAGTGTATTGGATT*A*C*G
c18739/f1p1/1392 AS	A*C*C*ACATCGGTCATCTGGGTCA*G*A*C
c13726/f1p1/1261 AS	C*T*T*GCATGAAATAAGTTCCTCCA*G*T*C
C4832/f1p61/1561 AS	G*C*A*TGAAAATTAAGGTCGTAGCAC*C*A*C
c21810/f1p2/1391 AS	C*A*C*AATTGAATTCAGATCCTTG*G*C*C
